# Relationships between Sleep, Athletic and Match Performance, Training Load, and Injuries: A Systematic Review of Soccer Players

**DOI:** 10.3390/healthcare9070808

**Published:** 2021-06-26

**Authors:** Filipe Manuel Clemente, José Afonso, Júlio Costa, Rafael Oliveira, José Pino-Ortega, Markel Rico-González

**Affiliations:** 1 Escola Superior Desporto e Lazer, Instituto Politécnico de Viana do Castelo, Rua Escola Industrial e Comercial de Nun’Álvares, 4900-347 Viana do Castelo, Portugal; 2 Instituto de Telecomunicações, Delegação da Covilhã, 1049-001 Lisboa, Portugal; 3 Centre for Research, Education, Innovation and Intervention in Sport, Faculty of Sport of the University of Porto, 4200-450 Porto, Portugal; jafonsovolei@hotmail.com; 4 Portugal Football School, Portuguese Football Federation, FPF, 1495-433 Cruz Quebrada, Portugal; julio.costa@fpf.pt; 5 Sports Science School of Rio Maior–Polytechnic Institute of Santarém, 2140-413 Rio Maior, Portugal; rafaeloliveira@esdrm.ipsantarem.pt; 6 Life Quality Research Centre, 2140-413 Rio Maior, Portugal; 7 Research Center in Sport Sciences, Health Sciences and Human Development, 5001-801 Vila Real, Portugal; 8 Faculty of Sports Sciences, University of Murcia, 30720 San Javier, Spain; josepinoortega@um.es; 9 BIOVETMED & SPORTSCI Research Group, University of Murcia, 30720 San Javier, Spain; markeluniv@gmail.com; 10 Department of Physical Education and Sport, University of the Basque Country, UPV-EHU, Lasarte 71, 01007 Vitoria-Gasteiz, Spain

**Keywords:** football, athletic performance, athletic injuries, load monitoring, sleep

## Abstract

The purpose of this systematic review was to summarize available evidence regarding the relationships between sleep and (i) athletic and match performance, (ii) training load, and (iii) injuries in soccer players. A systematic review of EBSCOhost (SPORTDiscus), PubMed, Cochrane Library, FECYT (Web of Sciences, CCC, DIIDW, KJD, MEDLINE, RSCI, and SCIELO) databases was performed according to the Preferred Reporting Items for Systematic Reviews and Meta-Analyses (PRISMA) guidelines. A total of 297 titles were identified, of which 32 met the eligibility criteria. Results revealed that soccer players are no exception for sleep inadequacy. Although there was inconsistency in the findings, some studies suggested that sleep restrictions in soccer negatively affected athletic and match performance while also increasing the number and severity of musculoskeletal injuries. On the other hand, inconsistent results were found between sleep and athletic and match performance, and training load in soccer players. Physiological responses (and their intensity) during drill-based games were not influenced by changes in sleep. The available evidence is inconsistent; however, it appears to suggest that poor sleep affects soccer players’ performance and increases the risk of injury. However, it remains important to study this complex relationship further.

## 1. Introduction

Sleep is essential to optimal health and restorative function [1], especially in athletes [2]. Thus, athletes and coaches recognize the relevance of sleep for supporting performance and recovery [3]. In an era of data monitoring, controlling the training process and factors affecting performance is crucial [4]. Much attention has been devoted to the evaluation of monitoring tools indicating signs of fatigue and/or the health status of athletes [5], and these include sleep monitoring [2]. The timing of preferred sleep and wake times affects the circadian rhythm, which affects sleep duration and quality and may affect athletic performance [6]. Among the general population, getting less than 8 h of sleep per night is associated with impaired cognitive performance, mood, and alertness, as well as increases in daytime sleepiness [7]. 

Workload and competition schedules influence the sleep/wake behaviors of young and adult soccer players at different competitive levels, especially during congested competitive periods [8,9]. Studies conducted on professional and non-professional young and adult soccer players [8,9,10] have shown that periods of intensified workloads increase the level of disturbance associated with sleep outcomes (e.g., decreased sleep duration and quality). The amount of sleep an athlete obtains interacts with training and competition schedules [2], and many athletes train later in the evening due to daily life commitments that need to be reconciled with their training schedules [11]. Adolescents have a higher physiological need for sleep (8–10 h per night) than adults (7–9 h per night) [12,13] and frequently experience delays in sleep onset and awakening. Soccer players report typical sleep durations within or above the recommended values for healthy adults [14], whereas swimmers report a sleep loss of 1.5 h because they often train early in the morning [15]. This suggests that the amount of sleep that athletes get depends on how training sessions are scheduled [16].

Sleep might be associated with the risk for injury and illness [2]. Studies have found that soccer players with shorter sleep duration and with low-quality (nonrestorative) sleep showed increased numbers and severities of musculoskeletal injuries [17,18]. In addition, Laux et al. [19] found that subjective sleep quality was associated with injury occurrence among elite soccer players in the month following an assessment, highlighting the role of sleep quality in the recovery process. Moreover, it has been hypothesized that short sleep durations impair athletes’ performance [2]. For instance, Souissi et al. [20] found that partial sleep deprivation in soccer players decreased their reaction time and squat jump performance, while peak and mean power were not affected. 

Thus, sleep seems to play an important role in constraint in athletic performance, training load, and injury risk in soccer players. These factors (performance, training load, and injury) are some of the determinants in soccer, and, naturally, understanding the well-being parameters that affect such elements can be determinants for maximizing performance and reducing exposure to injury. In this case, sleep may play an important role. However, there are different approaches performed in sleep analyses and their interactions with performance, training load, and injury. Thus, summarizing the evidence in a systematic review may help researchers and coaches to identify the state-of-the-art techniques and analyze the trends and possibilities for future studies and practical approaches. Specifically, determining the effects of regular sleep vs. sleep restrictions on athletic performance, training load, and injury risk may be crucial.

In summary, evidence indicates that workload and competition schedules—especially during congested competitive periods—are likely to impair athletes’ sleep, which can lead to injury, illness, and reduced performance. These factors highlight the need to prioritize sleep during congested periods, for instance, through sleep hygiene interventions. Therefore, the purpose of this systematic review was to summarize the evidence from studies on soccer players from any age group, sex, and competitive level who were exposed to monitorization of sleep. We aim to understand the effects of regular sleep vs. sleep restrictions on the main outcomes. In doing so, this study aims to assess outcomes concerning sleep-related measures in terms of performance, training load, and/or injury risk. 

## 2. Materials and Methods

The Cochrane Collaboration guidelines were followed to write the present systematic review [21]. The PRISMA (Preferred Reporting Items for Systematic Reviews and Meta-analyses) guidelines were followed [22]. The PECO (Population, Exposure, Comparator, Outcome) for this systematic review was defined as follows: (P) soccer players from any age group, sex, or competitive level; (E) exposure to sleep monitoring; (C) regular sleep vs. sleep deprivation or sleep loss (not mandatory); (O) athletic and match performance, training load, and/or athletic injuries occurrence. The protocol was published in INPLASY (International Platform of Registered Systematic Review and Meta-analysis Protocols) with the identification number of INPLASY202150029 and DOI 10.37766/inplasy2021.5.0029.

### 2.1. Eligibility Criteria

Table 1 presents the eligibility criteria.

### 2.2. Information Sources and Search

Electronic databases (EBSCOhost (SPORTDiscus), PubMed, Scielo, Cochrane, FECYT (Web of Sciences, CCC, DIIDW, KJD, MEDLINE, RSCI, and SCIELO)) were searched for relevant publications on 29 March 2021. Keywords and synonyms were entered in various combinations in the title and/or abstract, with two code lines for free terms (soccer OR football) AND (“sleep*”) AND one code line for MeSH terms (performance OR injur* OR load) NOT (“American football” OR “Australian football” OR rugby OR “Gaelic football”). After the search completed, and with the included articles being selected, a manual search was performed in their reference lists to retrieve additional studies that could fit our eligibility criteria. Two authors (J.A. and M.R.G.) independently performed the screening and analysis of the complete texts. Reference lists of the included studies were also searched, as well as corrections to the included studies, and the consultation of an independent expert.

### 2.3. Data Extraction

Data extraction used the Cochrane Consumers and Communication Review Group’s template [23]. Two authors (J.A. and M.R.G.) independently performed this step.

### 2.4. Data Items

The following information was extracted from the included original articles: (i) study design; (ii) goals of the study; (iii) characteristics of the participants (e.g., age, number, sex, competitive level); (iv) measures of sleep; (v) measures of athletic performance, match-running performance, training load, and athletic injuries. 

### 2.5. Methodological Assessment

The methodological assessment process was performed by two authors (M.R.G. and F.M.C.) using Downs and Black [24] assessment criteria for both randomized and non-randomized studies looking at studies eligible for inclusion. Each article was assessed based on 27 specific criteria (see Table 2). Each question is scored as 0 (poor quality) or 1 (good quality), with the exception of question 5 (“clear description of principal confounders”) that is scored from 0 (not satisfying) to 2 (fully satisfying) [25]. Therefore, a maximum of 28 points can be scored for each article. The quality of the article was classified based on the following thresholds [26]: (i) poor (<14 points); (ii) fair (14–18 points); (iii) good (19–23 points); and (iv) excellent (24–28 points). Any disagreement was discussed and solved by a consensus decision. Each item was evaluated using numerical characterization.

## 3. Results

### 3.1. Study Identification and Selection

**Figure 1:**
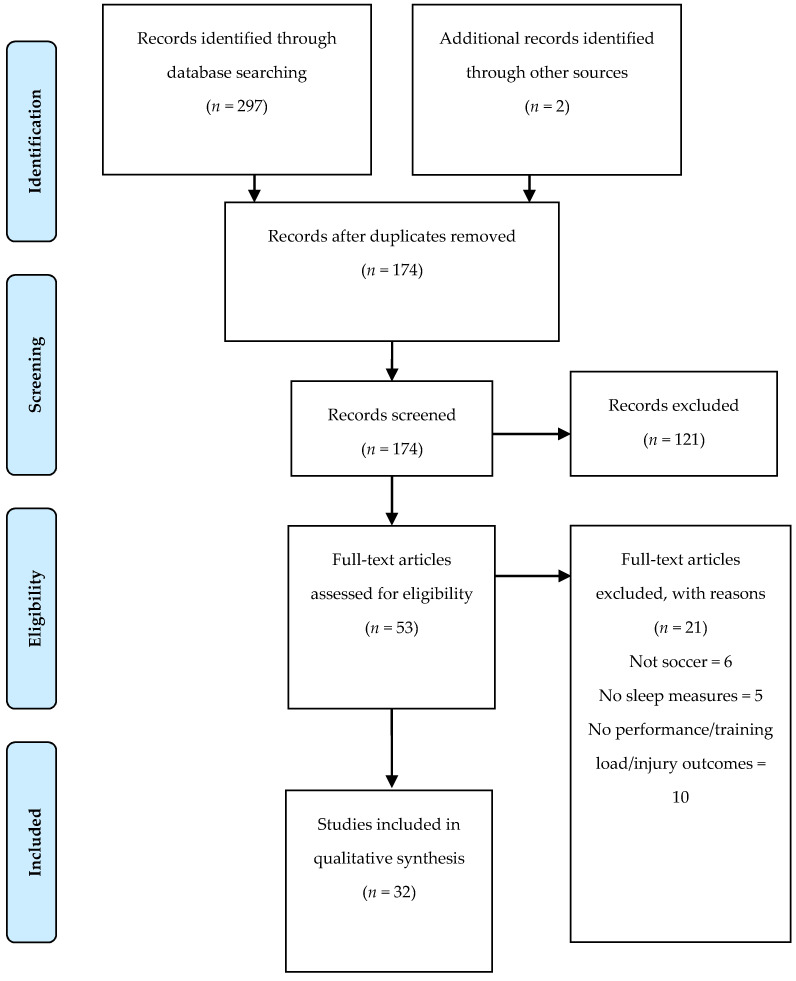
PRISMA flow diagram.

Searches retrieved 297 titles, plus two studies that were manually added. Duplicates (*n* = 125) were removed, and 174 studies were screened, of which 121 were removed. Of the 53 articles eligible for full text analysis, 32 were included in our review (Figure 1). Twenty-one were excluded based on the following reasons: (i) not in soccer (*n* = 6); (ii) no sleep measures (*n* = 5); and (iii) no performance/training load/injury outcomes (*n* = 10).

### 3.2. Study Characteristics

Thirty-two studies were included in the qualitative and quantitative analysis. Table 3 describes the characteristics of the studies. Only six studies analyzed under-18 soccer players; two studies did not describe age; and the remaining studies analyzed adult players. All studies were prospective, and 27 were a cohort type (see Table 3). There was one case report study [18], two randomized crossover studies [27,28], and two cross-sectional studies [29,30].

Five dimensions of analysis were considered: match analysis; athletic performance; training load; match and training load; and injury. For clarity, the match analysis included two studies that analyzed load from matches with relationship with sleep [31,32]. Athletic performance included 15 studies that applied different experimental designs through physical tests to analyze its influence on sleep [20,27,28,30,31,32,33,34,35,36,37,38,39,40,41]. Training load included six studies that observed training effects on sleep [42,43,44,45,46,47]. Match and training load included three studies that analyzed the effect of both dimensions on sleep. Eight studies were considered in the injury dimension because they analyzed the relationship between injury and sleep [17,18,19,29,48,49,50,51]. 

The most-used instrument to analyze sleep was actimetry, applied in twelve studies [8,9,10,17,18,20,33,38,39,40,41,47]. In addition, 25 studies included sleep logs/questionnaires to analyze its quantity and/or quality [10,17,18,19,27,28,29,30,31,32,33,34,35,36,40,41,42,43,44,45,46,48,49,50,51].

### 3.3. Methodological Quality

The methodological assessment and scores can be found in Table 2. Twenty-seven of the included articles [8,10,17,18,19,20,30,32,33,34,35,36,37,38,39,40,41,42,43,44,45,46,47,48,49,50,51] were classified with fair methodological quality, while five [9,27,28,29,31] were classified as good.

### 3.4. Results of Individual Studies: Sleep and Athletic and/or match Performance

The synthesis of results about sleep and athletic and/or match performance can be found in Table 4. Fifteen studies had data on athletic and or match performance [20,27,28,30,31,32,33,34,35,36,37,38,39,40,41]. Sleep restriction was applied in eight studies [20,27,28,31,37,38,39,40]. It was found that partial sleep deprivation negatively affects physical performance [20,27,28,31,38,39,40] and also perceived physical performance [37].

Traveling or jet leg was analyzed in three studies [33,35,41]. Regarding jet leg effects from international traveling, it was found that the first two days following affected physical performance and psychological variables [35]. The study of Fowler et al. [41] also reinforced such a finding in the day following air travel. Lastly, Fowler et al. [33] showed a positive effect by applying a light exposure and sleep hygiene program.

One study analyzed different physical tests and control if sleep quality affected the results [30] and found that sleep was not a major contributor for perceived exertion. 

### 3.5. Results of Individual Studies: Sleep and Training Load

The synthesis of results about sleep and training load can be found in Table 5. From 29 studies, six were considered in the training load dimension [42,43,44,45,46,47]. All studies included periods of training. Two of the studies [42,44] analyzed the sleep effects through small-sided games (SSG), while the others did not specify the exercises used in training. Five studies used wellness questionnaires to analyze sleep [42,43,44,45,46]. 

Two studies found negative correlations between perceived exertion and sleep quality [42,43]. One study did not find any correlation between sleep quality and physiological variables [44]. Two studies applied in female and male athletes, respectively, found that a higher quantity of sleep helped to improve subjective well-being variables [46] or training impulse scores in the Ramadan Fasting period [47]. In addition, Watson and Brickson [45] also found that decreasing sleep quantity could be related to a negative effect in higher training load levels.

### 3.6. Results of Individual Studies: Sleep and Training/Match Performance

The synthesis of results about match and training load with sleep can be found in Table 6. From 29 studies, three studies analyzed a period of training and matches [8,9,10]. All studies analyzed [8,9,10] variations of sleep duration and the relationships between match/training with sleep measures through correlations. One study found a correlation between sleep duration and session rated of perceived exertion [8], while the other two studies showed that sleep variability could present a major role to interpret results and reinforced an individual approach since training and match could negatively impact some athletes [9,10].

### 3.7. Results of Individual Studies: Sleep and Injury Risk 

The synthesis of results about sleep and injuries can be found in Table 7. From 29 studies, eight quantified the number of injuries for the period of the analysis through logs or questionnaires [17,18,19,29,48,49,50,51]. One study analyzed repetitive head impacts [51] and suggested that good sleep quality and quantity may avoid such head impacts, while two studies analyzed musculoskeletal injuries with common mental disorders [29,49]. Gouttebarge et al. [29] showed that higher sleep disturbance could be associated with joint or muscle injury, while in opposition, Kiliç et al. [49] found no relationship between sleep disturbance and musculoskeletal injuries [49]. 

The remaining five studies analyzed the number of injuries and its relationship with sleep quality or quantity [17,18,19,48,50]. From these, one study showed that the Ramadan period which impact sleep schedule and can increase the number of injuries [48]. In addition, it was found that small-field tournaments showed lower values of sleep quantity in the night before the match where higher injury was revealed [50]. The other three studies were in line by showing that bad sleep quality or quantity is associated with a higher risk of injury [17,18,19]. 

**Table 2:** Methodological assessment of the included studies.

Reference	Reporting										Ext. Validity			Int. Validity							Int. Validity-Cofounding						Power	Score
	1	2	3	4	5	6	7	8	9	10	11	12	13	14	15	16	17	18	19	20	21	22	23	24	25	26	27	Final
Abbott et al., [31]	1	1	1	1	2	1	1	0	0	1	1	0	1	0	0	1	1	1	0	1	1	1	1	0	1	0	1	20
Abedelmalek et al., [38]	1	1	1	1	2	1	1	0	0	0	0	0	0	0	0	1	1	1	0	1	1	1	1	0	1	0	0	16
Abedelmalek et al., [39]	1	1	1	1	2	1	1	0	0	0	1	0	0	0	0	1	1	1	0	1	1	1	1	0	1	0	0	17
Ajjimaporn et al., [27]	1	1	1	1	2	1	1	0	0	0	1	1	0	0	0	1	1	1	0	1	1	1	1	0	1	1	1	20
Aziz et al., [32]	1	1	1	1	2	1	1	0	0	1	0	1	1	0	0	1	1	1	0	1	1	1	0	0	1	0	0	18
Baati et al., [40]	1	1	1	1	2	1	1	0	0	0	1	0	0	-	-	1	-	1	-	1	1	1	-	-	1	0	0	15
Chamari et al., [48]	1	1	1	1	2	1	1	0	1	0	1	0	1	-	-	1	-	1	-	1	1	1	-	-	1	1	0	18
Clemente et al., [42]	1	1	1	1	2	1	1	0	0	0	1	0	1	-	-	1	-	1	-	1	1	1	-	-	1	0	0	16
Clemente et al., [43]	1	1	1	1	2	1	1	0	0	1	1	0	1	-	-	1	-	1	-	1	1	1	-	-	1	0	0	17
Fowler et al., [41]	1	1	1	1	2	1	1	0	0	1	1	0	0	-	-	1	-	1	-	1	1	1	1	-	1	0	0	17
Fowler et al., [33]	1	1	1	1	2	1	1	0	0	0	1	0	1	0	0	1	0	1	1	1	1	1	1	0	1	0	0	18
Gouttebarge et al., [29]	1	1	0	1	2	1	1	0	0	1	1	1	0	0	1	1	1	1	0	1	1	1	-	1	1	1	1	21
Güvenç et al., [34]	1	1	1	1	2	1	1	0	0	0	1	0	1	-	-	1	-	1	-	1	1	1	-	-	1	0	0	16
Haddad et al., [30]	1	1	0	1	2	1	1	0	1	0	1	0	1	-	-	1	-	1	-	1	1	1	-	-	1	1	0	17
Hill et al., [35]	1	1	1	1	2	1	1	1	0	0	1	0	1	-	-	1	-	1	-	1	1	1	-	-	1	0	0	17
Kiliç et al., [49]	1	1	0	1	2	1	1	0	0	0	1	1	1	-	-	1	-	1	-	1	1	1	-	-	1	1	1	18
Krutsch et al., [50]	1	1	1	1	2	1	1	0	0	0	1	0	0	-	-	1	-	1	-	1	1	1	-	-	1	1	0	16
Laux et al., [19]	1	1	0	1	2	1	1	0	1	0	1	1	1	-	-	1	-	1	-	1	1	1	-	-	1	0	0	17
Levitch et al., [51]	1	1	0	1	2	1	1	0	0	0	1	1	0	-	-	1	-	1	-	1	1	1	-	-	0	0	0	14
Meckel et al., [36]	1	1	1	1	2	1	1	0	0	0	0	0	1	-	-	1	-	1	-	1	1	1	-	-	1	0	0	15
Nédélec et al., [18]	1	1	0	1	2	1	1	0	0	0	1	0	1	-	-	1	-	1	-	1	1	1	-	-	1	0	0	15
Pallesen et al., [28]	1	1	1	1	2	1	1	1	0	0	1	0	1	0	0	1	1	1	1	1	1	1	1	0	1	0	1	21
Selmi et al., [44]	1	1	1	1	2	1	1	0	0	0	1	0	1	-	-	1	-	1	-	1	1	1	-	-	1	0	0	16
Silva et al., [17]	1	1	1	1	2	1	1		0	0	0	0	1	0	0	1	1	1	0	1	1	1	-	0	1	0	0	16
Souissi et al., [20]	1	1	1	1	2	1	1	0	0	0	1	0	1	0	0	1	1	1	0	1	1	1	1	0	1	0	0	18
Watson & Brickson [45]	1	1	1	1	2	1	1	0	0	1	0	0	1	-	-	1	-	1	-	1	1	1	-	-	1	0	0	16
Watson et al., [46]	1	1	1	1	2	1	1	0	0	0	0	0	1	-	-	1	-	1	-	1	1	1	-	-	1	0	0	15
Wilson et al., [47]	1	1	1	1	2	1	1	0	0	0	1	0	0	-	-	1	-	1	-	1	1	1	-	-	1	0	0	15
Zerguini et al., [37]	1	1	0	1	2	1	1	1	0	0	1	0	1	-	-	1	-	1	-	1	1	1	-	-	1	0	0	16
Costa et al., [9]	1	1	1	1	2	1	1	1	1	0	1	1	0	-	-	1	-	1	-	1	1	1	-	-	1	1	0	19
Figueiredo et al., [8]	1	1	1	1	2	1	1	0	0	0	1	1	0	-	-	1	-	1	-	1	1	1	-	-	1	1	0	17
Costa et al., [10]	1	1	1	1	2	1	1	0	0	0	1	1	0	-	-	1	-	1	-	1	1	1	-	-	1	1	0	17

**Note:** A detailed explanation of the domains and categories, together with instructions for the analysis, can be consulted in the original article [24].

**Table 3:** Characteristics of individual studies.

Study	N/Age/Sex	Study Design	Dimension Analysis	Instruments and Outcomes for Sleep	Instruments/Tests and Outcomes for Performance, Training Load Or Injury
Abbott et al., [31]	N: 9 Age: 21 ± 5 Sex: M	Prospective cohort	Match analysis	Sleep log: total sleep time. Sleep quality (1–5 scale).	RPE (CR-10). CMJ.
Abedelmalek et al., [38]	N: 36 Age: 21.3 ± 0.5 Black Tunisian Age: 22.1 ± 2.2 White Tunisian Age: 22.2 ± 1.3 South African Sex: M	Prospective cohort	Athletic performance	Actimetry (Actiwatch sleep and Activity software V5.32; Cambridge Neurotechnology; Cambridge, UK): total sleep duration, sleep latency; sleep efficiency and mean activity score (the average value of the activity counts per epoch over the assumed sleep period).	30 s Wingate test: Peak power Mean power Fatigue index
Abedelmalek et al., [39]	N: 12 Age: 21.2 ± 1.2 Sex: M	Prospective cohort	Athletic performance	Actimetry (Actiwatch sleep and Activity software V5.32; Cambridge Neurotechnology, Cambridge, UK): total sleep duration, sleep, sleep efficiency.	30 s Wingate test: Peak power Mean power Fatigue index.
Ajjimaporn et al., [27]	N: 11 Age: 20 ± 1 Sex: M	Prospective randomized crossover	Athletic performance	Total sleep duration.	RPE (CR-20). Auditory reaction time. RAST: max power, min power, average power, fatigue index. Isometric leg strength test.
Aziz et al., [32]	N: 13 Age: ND (under 23) Sex: M	Prospective cohort	Match analysis	Time to bed and waking. Daytime nap times. Epworth Sleepiness Scale questionnaire: levels of daytime sleepiness.	Brunel Mood Scale. Visual analogue scale. Profile of mood state. Perceived readiness questionnaire. S-RPE (CR-10). 5 Hz GPS (MinimaxX 2.5; Catapult Innovations, Melbourne, Australia): total distance, low-speed (<8.0 km·h−1), moderate-speed (8.0–18.0 km·h^−1^) and high-speed running (>18.0 km·h−1); HR monitor strap (T-31, Polar Oy, Kempele, Finland): HRavg, HRmax. Blood lactate (LactatePro, Arkray Inc., Kyoto, Japan).
Baati et al., [40]	N: 10 Age: 22.8 ± 1.3 Sex: M	Prospective cohort	Athletic performance	Wrist actigraphy (Actiwatch sleep and Activity software, version 5.32; Neurotechnology, Cambridge, UK): sleep latency, sleep efficiency, end of sleep, start of sleep. Hooper Index: sleep quality.	Egocentric Distance Estimation 3 distances (15, 25, and 35 m) before and after repeated cycling (Monark 894E, Stockholm, Sweden). POMS questionnaire. Hooper Index: stress, fatigue, muscle soreness.
Chamari et al., [48]	N: 42 Age: 24 ± 4 Sex: M	Prospective cohort	Injury	Hooper Index: sleep quality.	RPE (CR-10 scale): Weekly training load Weekly training strain Weekly training duration. Hooper index: stress, fatigue, muscle soreness. Injury rate: Rate of contact injury Rate of non-contact injury Rate of contact injury during matches Rate of overuse injury during matches Rate of contact injury during training Rate of overuse injury during training Number of injuries per 4-week periods Total monthly training and match exposure.
Clemente et al., [42]	N: 10 Age: 19.8 ± 1.6 Sex: M	Prospective cohort	Training load	Hooper Index: sleep quality.	HRavg (Polar H7, Polar Electro, OY, Kempele, Filand). GPS (JOHAN Sports, Noordwijk, The Netherlands): total distance (m); running distance at 14–19.9 km/h sprinting distance at >20.0 km/h; total number of accelerations >2 m/s^2^; RPE (CR-10 scale). Hooper Index: stress, muscle soreness and fatigue.
Clemente et al., [43]	N: 35 Age: 25.7 ± 5.0 Sex: M	Prospective cohort	Training load	Hooper Index: sleep quality.	RPE (CR-10 scale). Hooper Index: stress, muscle soreness and fatigue.
Costa et al., [9]	N: 20 Age: 25.2 ± 3.1 Sex: F	Prospective cohort	Training and match analysis	Wrist actigraphy (Actigraph LLC wGT3X-BT, Pensacola, FL, USA): sleep time, time in bed, wake-up time, sleep onset time, wake after sleep onset, sleep fragmentation index, latency and sleep efficiency.	s-RPE (CR-10). 18 Hz GPS units (STATSports Apex, Northern Ireland): total distance training and match exposure time high-speed running
Costa et al., [10]	N: 34 Age: 20.6 ± 2.3 Sex: F	Prospective cohort	Training and match analysis	Hooper Index: sleep quality. Accelerometer (Actigraph LLC wGT3XBT, Pensacola, FL, USA): sleep duration and sleep efficiency.	s-RPE (CR-10). TRIMP. HR (Firstbeat Sports, Jyväskylä, Finland): %HRmax and HR exercise. Hooper Index: fatigue, stress and muscle soreness.
Figueiredo et al., [8]	N: 18 Age: 14.8 ± 0.3 Sex: M	Prospective cohort	Training and match analysis	Accelerometer (Actigraph LLC wGT3X-BT, Pensacola, FL, USA): sleep duration, sleep efficiency,	s-RPE (CR-10).
Fowler et al., [41]	N: 10 Age: 23.9 (22.2–25.6) Sex: M	Prospective cohort	Athletic performance	Actigraphy watches (ReadiBand, Fatigue Science, Honolulu, HI, USA)—sleep duration, latency, efficiency, number of awakenings, and duration of awakenings. Hooper Index: sleep quality.	CMJ—linear position transducer (GymAware, Kinetic Performance Technologies, Canberra, Australia): jump height, peak power, and peak velocity. Yo-Yo Intermittent Recovery level 1 test (YYIR1). HR, HRmax, HRR (Polar Team2, Polar Electro, Kempele, Finland). Cortisol (ELISA, Demeditec Diagnostics, Kiel, Germany). Oxygen saturation (Nonin 4000 Avant Bluetooth Pulse Oximeter, Nonin, North Plymouth, MN, USA) Hooper Index: stress, fatigue and muscle soreness. S-RPE. Physical feeling. Brunel Mood Scale.
Fowler et al., [33]	N: 20 Age: 21.9 ± 3.6 Sex: M	Prospective cohort	Athletic performance	Actigraphy (Actiwatch-64; Philips Respironics, Bend, OR, USA): time in bed; sleep onset; sleep offset; sleep duration; sleep efficiency. Subjective sleep quality (Liverpool John Moore’s University jet lag questionnaire).	CMJ: jump height, peak power, and peak velocity 20 m sprint, T test, Yo-Yo Intermittent Recovery Level 1. Brunel Mood Scale. s-RPE. Physical feeling.
Gouttebarge et al., [29]	N:540 Age: 26.7 ± 4.4 Sex: M	Prospective cross-sectional	Injury	Sleeping disturbance—PROMIS (short form).	3 Musculoskeletal Questionnaire: total number of severe musculoskeletal injuries; total number of severe joint injuries; and total number of severe muscle injuries during a professional soccer career. Number of Surgeries.
Güvenç et al., [34]	N:16 Age:17.4 ± 1.2 Sex: M	Prospective cohort	Athletic performance	Sleep duration.	Modified 20 m shuttle run test: peak running distance, peak running time, peak running velocity, running velocity at 4.0 mmol·L^−1^, peak LA, peak HR, peak RPE Blood Lactate (LA) YSI 1500 Sport Lactate Analyzer (Yellow Springs Inst., Yellow Springs, OH, USA) HR monitors (S610i, Polar Electro Oy, Kempele, Finland): HRmax, HRavg, RPE (CR-20) LA/HR/RPE: Resting: at 8 km·h−1 at 10 km·h−1 at 11 km·h−1 at 12 km·h−1
Haddad et al., [30]	N: 17 Age: 17–19 Sex: M	Prospective cross-sectional	Athletic performance	Hooper Index—sleep quality	Heart rate (Polar S-800i, Polar Electro, Finland): HRmax, RHR, HRres. RPE (CR-10 scale). Hooper Index: stress, muscle soreness, and fatigue.
Hill et al., [35]	N: 7 Age: 22 ± 2 Sex: F	Prospective cohort	Athletic performance	Sleep duration. Sleep quality (1-to-5 non-validated scale).	Mood state—POMS questionnaire: tension/anxiety, depression/dejection, anger/hostility, vigor/activity, fatigue/inertia, and confusion/bewilderment. Grip strength: handgrip dynamometer (Asimov Engineering, Los Angeles, CA, USA).
Kiliç et al., [49]	N: 262 Age: 27 ± 5 Sex: M	Prospective cohort	Injury	Sleep disturbance—PROMIS.	Musculoskeletal disorder injury.
Krutsch et al., [50]	N: 423 M (22.9 ± 2.6) N: 271 F (22.2 ± 2.3)	Prospective cohort	Injury	Sleep quality: sleep duration, any sleep interruptions during the night, feeling tired in the morning of the match day	Total playing time in the tournament. Mean playing time per player in the tournament. Frequent training sessions before the tournament. Participation in a structured warm-up routine before the matches. Playing in a soccer club Traumatic Injury Prevalence per player Overuse injuries Prevalence per player
Laux et al., [19]	N: 22 Age: 25.8 ± 5 Sex: M	Prospective cohort	Injury	RESTQ-Sport questionnaire: sleep quality.	RESTQ-Sport questionnaire: general stress, emotional stress, social stress, conflicts/pressure, fatigue, lack of energy, physical complaints, success, social recovery, physical recovery, general well-being, disturbed breaks, emotional exhaustion, injury, being in shape, personal accomplishment, self-efficacy, self-regulation.
Levitch et al., [51]	N: 50 Age: 18 Sex: F	Prospective cohort	Injury	Pittsburgh Sleep Quality Index: quality, latency, duration, efficiency, disturbances, sleeping medication, daytime dysfunction.	HeadCount Web-based questionnaire: HeadCount-12m inquired about soccer activity during the prior 12 months; HeadCount-2w inquired about soccer activity during the prior two weeks.
Meckel et al., [36]	N: 18 Age: 15.1± 0.9 Sex: M	Prospective cohort	Athletic performance	Sleep (hours/day)	Vertical jump test; Agility test; Speed endurance test; Sprint test; Endurance test
Nédélec et al., [18]	N: 1 Age: 31 Sex: M	Prospective cohort case report	Injury	Wrist activity monitor (Actisleep, TheActigraph, Pensacola, FL, USA): bed time, sleep onset latency, total sleep time, sleep efficiency. Sleep quality using a visual 10-point analogue scale.	s-RPE (CR-10). Injuries number.
Pallesen et al., [28]	N: 19 Age: 16.5 ± 1.3 Sex: M	Prospective randomized crossover	Athletic performance	Horne-Ostberg Morningness-Eveningness Questionnaire: sleep habits, sleepiness, preferred time for performing activities. Sleep time. Alert/sleep: Karolinska Sleepiness Scale.	Juggling test; Dribbling test (20 and 40 m sprints, 0 m sprint with directional change); Ball control (trapping); Continuous kicking.
Selmi et al., [44]	N: 20 Age: 25.1 ± 1.0 Sex: M	Prospective cohort	Training load	Hooper Index: sleep quality	Hooper Index: fatigue, stress, and muscle soreness. Total quality recovery questionnaire. VAMEVAL test: HRmax. RPE (CR-10 scale).
Silva et al., [17]	N: 23 Age: 26.5 ± 5.2 Sex: M	Prospective cohort	Injury	Self-reporting sleep diaries. Actiwatch 2 wrist activity monitor actigraph (Philips Respironics, Andover, MA, USA): time awake, sleep duration, WASO (Wake After Sleep Onset), sleep efficiency, and sleep latency.	Number of injuries, the injury severity, and the absence time.
Souissi et al., [20]	N: 14 Age: 23.57 ± 1.98 Sex: M	Prospective cohort	Athletic performance	Actimetry (Actiwatch sleep and Activity software v5.32; Cambridge Neurotechnology; UK): sleep duration.	Squat jump test (Optojump, Italy). 30 s Wingate test (Monark 894E, Stockholm, Sweden). Index of motor performance: reaction time.
Watson and Brickson [45]	N: 65 Age: 15.5 ± 1.6 Sex: F	Prospective cohort	Training load	Daily ratings of sleep quality. Sleep duration.	RPE (CR-10 scale) Daily ratings of fatigue, mood, soreness, stress.
Watson et al., [46]	N: 75 Age: 15.5 ± 1.6 Sex: F	Prospective cohort	Training load	Daily ratings of sleep quality. Sleep duration.	s-RPE (CR-10 scale). Daily ratings of fatigue, mood, soreness, stress. Injuries numbers, locations, and types.
Wilson et al., [47]	N: 14 Age: 25 ± 3 Sex: M	Prospective cohort	Training load	Actimeter watch (Cambridge Neurotechnology, Cambridge, UK): time that they went to bed and woke-up, bedtimes, wake-up times.	20 m progressive shuttle run: VO_2max_ (Polar Team System, Kempele, Finland). Relative HR zones and TRIMP were calculated.
Zerguini et al., [37]	N: 55 Age: ND Sex: M	Prospective cohort	Athletic performance	Sleep hours and Sleep quality	Explosive leg power: standing vertical jump. Agility: four-line test. Dribbling test: test of soccer skill. Speed and acceleration were obtained by photoelectric cells during a 20 m sprint from a standing start. Endurance: 12 min run, with recovery HR monitored for 5 min.

N: number; M: male; F: female; ND: non-described. PROMIS: Patient-Reported Outcomes Measurement Information System; RPE: rated perceived exertion; s-RPE: session rated perceived exertion; RAST: Running-based Anaerobic Sprint Test; GPS: global positioning system; HR: heart rate; HRavg: heart rate average; HRmax: heart rate maximal; HRR: heart rate recovery; CMJ: counter movement jump; TRIMP: training impulse.

**Table 4:** Results concerning sleep and athletic and/or match performance.

Study	Aim	Results	Findings
Abbott et al., [31]	To investigate the cognitive, physical, and perceptual effects of sleep restriction in soccer players following a night match.	CMJ decreased by ~8% after the match with and without sleep restriction.	Sleep restriction (~6 h of sleep) following a nighttime soccer match does not impair CMJ performance, subjective wellbeing, or cognitive function in the following morning.
Abedelmalek et al., [38]	To access the effect of PSD and racial variation on muscle power and fatigue during a 30-s Wingate test.	There was a significantly greater decrease in PP and MP (*p* < 0.001) after the PSD in South Africans compared with black Tunisians (*p* < 0.05) and white Tunisians (*p* < 0.05); values for South Africans, White Tunisians, and Black Tunisian. Fatigue index was unaffected by either sleep deprivation or racial variation.	4 h of PSD at the end of the night affect performance during the Wingate test at 18:00 h more in South Africans than Tunisians. These results suggest a greater vulnerability of South Africans to sleep deprivation.
Abedelmalek et al., [39]	To evaluate the effect of time of day and PSD on short-term maximal performance and level of IL-6 in trained subjects.	PP and MP improved significantly from the morning to the afternoon after NNS (*p* < 0.05) and from the afternoon to the morning after PSD (*p* < 0.05). Compared to NNS, PP and MP were not affected by PSD the following morning. However, there was a significant decrease in PP and MP (*p* < 0.001) after the PSD at 18:00 h.	A short-term high-intensity exercise may increase the interleukin-6 concentrations in the morning and the afternoon. Moreover, IL-6 remained elevated during the recovery period in the afternoon after the PSD at the end of the night.
Ajjimaporn et al., [27]	To examine effects of a 20-min nap following 3 h of sleep deprivation on brain wave activity, auditory reaction time, the RAST, leg muscle strength, and the RPE in male college soccer players.	The sleep deprivation demonstrated an increase in the MP of delta waves over the frontal area and a decrease in the mean power of alpha waves over the parietal area compared to the normal sleep. The nap and the sleep deprivation showed an increase in auditory reaction time compared with those in the normal sleep. The sleep deprivation demonstrated a decrease in the running-based anaerobic sprint test compared to the normal sleep, whereas the nap has partially reversed only minimal power and average power of the running-based anaerobic sprint test. The nap showed a recovery effect on leg muscle strength, but not on the rating of perceived exertion compared with the sleep deprivation.	Getting 3 h of sleep a night had negative effects on anaerobic performance test, muscle strength, and fatigue in the afternoon (16:00 h) of the following day. A 20min nap after sleep deprivation did not entirely reverse the negative impact of sleep deprivation on soccer performance.
Aziz et al., [32]	To examine the effects of RF on physical activity profile of soccer players via the satellite GPS during a 90 min match played.	In RF condition, players covered a lower total distance (by 12.8 ± 5.8%), and covered less distance within the moderate (22.4 ± 12.0%) and high-speed (35.5 ± 20.1%) zones (all *p* < 0.025). Players accomplished a lower relative speed throughout the most of the match in RF compared to control condition (*p* < 0.008). Blood glucose, blood lactate, and HR of the players during match in RF and control conditions were equivalent (all, *p* > 0.05), although RPE was higher before, during, and post-match in the RF vs. control conditions (*p* > 0.05).	Players’ physical activity profile during a soccer game was adversely affected by RF which include 6.5 h of sleep and 100 min of daily nap, and this negative impact was already observed in the initial stages of the match.
Baati et al., [40]	To investigate the effects of intensive effort on egocentric distance perception according to different angles of view after SDB or SDE of the night and after a NNS.	For 35 m, distance estimation was better during NNS compared to SDB and SDE for the front and the two side angles either before or after RS. Concerning 25 m, distance estimation was better after compared to before repeated cycling for the front angle during the NNS session (*p* < 0.05). For 15 m, distance estimation was better during NNS than SDB and SDE for the front and both side angles after repeated cycling (*p* < 0.05). All Hooper index categories and POMS denoted better results after NNS compared to SBD and SDE (*p* < 0.05).	PSD negatively affected the estimation of the egocentric distance for the three angles of view either at rest or after repeated cycling exercise.
Fowler et al., [41]	To examine the effects of simulated air travel on physical performance.	Sleep quantity and quality were significantly reduced during international simulated travel compared with control group and domestic simulated travel (*p* < 0.01). Yo-Yo Intermittent Recovery level 1 test performance was significantly reduced in the PM following day during international simulated compared with control group and domestic simulated travel (*p* < 0.01), where performance remained unchanged (*p* > 0.05). Compared with baseline, physiological and perceptual responses to exercise, and mood states, were exacerbated following the international simulated travel (*p* < 0.05).	Attenuated intermittent-sprint performance following simulated international air travel may be due to sleep disruption during travel and the subsequent exacerbated physiological and perceptual markers of fatigue.
Fowler et al., [33]	To assess the efficacy of a combined light exposure and sleep hygiene intervention to improve team-sport performance following eastward long-haul transmeridian travel.	Magnitude-based inference and standardized effect-size analysis indicated there was a very likely improvement in the mean change in countermovement jump peak power (effect size 1.10 ± 0.55), and likely improvement in 5 m (0.54 ± 0.67) and 20 m (0.74 ± 0.71) sprint time in intervention group compared with control group across the 4 days post-travel. Sleep duration was most likely greater in intervention group both during travel (1.61 ± 0.82) and across the 4 nights following travel (1.28 ± 0.58) compared with control group. Finally, perceived mood and motivation were likely worse (0.73 ± 0.88 and 0.63 ± 0.87) across the 4 days post-travel in control group compared with intervention group.	Combined light exposure and sleep hygiene improved speed and power but not intermittent-sprint performance up to 96 h following long-haul transmeridian travel. The reduction in sleep disruption during and following travel is a likely contributor to improved performance.
Güvenç et al., [34]	To examine the effects of RF on body composition, aerobic exercise performance, blood lactate, heart rate, and perceived exertion in regularly trained young soccer players.	Although RPE at submaximal workloads increased during RF (*p* < 0.05), blood lactate and HR had decreased by the end of RF (*p* < 0.05). Peak running performance and running velocity at anaerobic threshold also improved by the end of RF (*p* < 0.05).	Regular training regimen, body fluid balance, daily energy intake, and sleep duration (8.6–8.8 h) are maintained during RF; it does not have detrimental effects on aerobic exercise performance or body composition in young soccer players.
Haddad et al., [30]	To assess Hooper Index effects on RPE during a 10 min submaximal exercise training session.	No significant correlations were resulted between RPE-10 min and Hooper’s Index in all athletes.	The results suggest that fatigue, stress, muscle soreness, and sleep are not major contributors of RPE during traditional soccer training without excessive training loads.
Hill et al., [35]	To evaluate the effects of jet lag on factors associated with sport performance	Vigor was reduced, *p* < 0.05, on Days 1 and 2 in Taiwan. Fatigue was increased on Day 1, *p* < 0.05, Day 2 (non-significant), and Day 3 (non-significant). Total mood disturbance was elevated, *p* < 0.05, on Days 1 and 2. Grip strength measured the first 2 days in Taiwan was lower, *p* < 0.05, than the baseline value. Number of hours spent sleeping differed, *p* < 0.05, from baseline on the 2nd night in Taiwan, with the athletes apparently trying to catch up on lost sleep. There was no significant deterioration in sleep quality, despite an apparent trend toward poorer quality from the 1st through 4th days at the destination.	This finding suggests that, despite their high level of motivation and preparation, even elite athletes may suffer psychological decrements after rapid transmeridian travel. Performance-related variables that were evaluated were seen to return quite quickly to pretravel levels. The iceberg mood state profile of the elite athletes and the fit physical educators was re-established by the 4th day at the destination.
Meckel et al., [36]	To examine the effect of the RF on performance capacities, dietary habits, and the daily behavioral patterns in adolescent soccer players.	There were no significant differences in total daily sleeping hours (8.6 ± 0.7 h/day vs. 8.6 ± 0.5 h/day, *p* = 0.80) between RF and a regular month.	The decrease in performance does not necessarily relate to changes in caloric intake and sleeping hours during the fast.
Pallesen et al., [28]	To investigate the effects of sleep deprivation on soccer skills (habitual sleep and 24 h sleep deprivation).	The results revealed a negative effect of sleep deprivation on the continuous kicking test. On one test, 30 m sprint with directional changes, a significant condition test repetition interaction was found, indicating a steeper learning curve in the sleep-deprived condition from Test 1 to Test 2 and a steeper learning curve in the rested condition from Test 2 to Test 3.	Negative effects of sleep deprivation on soccer skills were partly supported by the data and that more pronounced effects would be expected in a soccer match. Greater negative sleep impact over repetitive performance was not supported by these data.
Souissi et al., [20]	To evaluate the effects of caffeine ingestion and partial sleep deprivation at the end of night on cognitive and physical performance.	Results showed that reaction time squat jumps were affected by PSD, even though peak power, mean power and SJ were not affected on the following day. However, both simple and choice reaction times were significantly poorer during PSD in comparison with NNS (*p* < 0.05 and *p* < 0.001, respectively).	PSD decreases reaction time and squat jumps, but peak and mean power were not affected.
Zerguini et al., [37]	To analyze effects of RF on Muslim soccer athletes.	Nearly 70% of the players thought that their training and performance were adversely affected during the fast where players’ sleep was reduced by 30 min.	The phase shift of food intake and disruption of sleep patterns affect actual and perceived physical performance. Islamic athletes need to explore strategies that will maximize performance during Ramadan.

CMJ: counter movement jump; PSD: partial sleep deprivation; PP: peak power; MP: mean power; IL-6: interleukin-6; RAST: running-based anaerobic sprint test; RPE: rated perceived exertion; RF: Ramadan Fasting; GPS: global positioning system; HR: heart rate; NNS: normal sleep night; SDB: sleep deprivation at the beginning of the night; SDE: sleep deprivation at the end of the night.

**Table 5:** Results concerning sleep and training load.

Study	Aim	Results	Findings
Clemente et al., [42]	To test the associations between wellness and internal and external load variables during two intermittent SSGs.	Large and negative correlations were found between sleep quality and RPE (−0.64, (−0.88; −0.14)) and total accelerations (−0.64, (−0.88; −0.13)) during 6 × 3′ small-sided games.	Sleep was also negatively and largely correlated with RPE during shorter bouts. Thus, coaches should adopt supplementary monitoring methods to avoid erroneous classifications of load rated by players with low sleep quality.
Clemente et al., [43]	To assess differences of playing position on s-RPE and Hooper Index across two different training microcycles (1 vs. 2 competitive games) and to examine the relationship between s-RPE and Hooper Index across an entire season.	Sleep quality was similar in the two different microcycles. Significative correlations between s-RPE and sleep were found (*p* = −0.109) in 2-matches microcycles.	Sleep was also negatively correlated with s-RPE, especially in microcycles with two matches, thus suggesting that congested periods that increase stress and muscle soreness are more sensitive to affect sleep quality.
Selmi et al., [44]	To assess the influence of well-being indices (sleep, stress, fatigue, and muscle soreness) and the total quality of recovery on technical and physiological measures during soccer SSGs.	No significant correlations were found between well-being indices, total quality recovery, and physiological parameters.	Physiological responses during SSGs (HR and Lactate) and its intensity were not influenced by the variability in sleep.
Watson and Brickson [45]	To determine whether acute TL and sleep are independent predictors of subjective well-being and whether sleep mediates the influence of acute TL on subjective well-being in female youth athletes.	Sleep duration mediated a significant portion of the effect of TL on mood (26.8%, *p* < 0.001), fatigue (12.6%, *p* < 0.001), and stress (24.5%, *p* < 0.001)	Among female youth athletes, decreased sleep duration and increased TL are independently associated with impairments in subjective well-being. In addition, decreased sleep mediates a significant portion of the negative effect of increases in TL on subjective well-being.
Watson et al., [46]	To determine whether acute TL and sleep are independent predictors of subjective well-being and whether sleep mediates the influence of acute TL on subjective well-being in female youth athletes.	Average sleep duration was 7.9 ± 1.4 h during the study period. Sleep duration mediated a significant portion of the effect of TL on mood (26.8%, *p* < 0.001), fatigue (12.6%, *p* < 0.001), and stress (24.5%, *p* < 0.001).	Increased sleep was significantly associated with improved fatigue, mood, and stress. In fact, a significant portion of the effect of TL on subjective well-being was because of TL on sleep.
Wilson et al., [47]	To establish how the timing of sleep was influenced by fasting diurnal requirements and how training practices were altered in professional soccer players by comparing behavior over the RF month to that displayed in the four weeks immediately following.	The TL, as indicated by training impulse scores, did not vary between RF and the following four weeks, although the duration of training sessions was shortened after two weeks of RF. Duration of sleep was prolonged during RF by 99 min on average.	A significant change in the timing of sleep was the main way that a group of professional soccer players adjusted to cope with the RF. Thus, influence of RF on daily activities is more a matter of chronobiology than calorie restriction.

RPE: rated perceived exertion; s-RPE: session rated perceived exertion; HR: heart rate; TL: training load; RF: Ramadan Fasting; SSGs: small-sided games.

**Table 6:** Results concerning sleep and training/match performance.

Study	Aim	Results	Findings
Figueiredo et al., [8]	To describe habitual sleep and nocturnal cardiac autonomic activity and their relationship with training/match load in male youth soccer players during an international tournament	During the five nights, 8 to 17 players slept less than <8 h and only one to two players had a sleep efficiency <75%. Players’ sleep duration CV ranged between 4 and 17%. A moderate within-subjects correlation was found between s-RPE and sleep duration.	The present findings suggest that youth soccer players slept less than the recommended amount during the international tournament, and sleep duration was negatively associated with training/match load.
Costa et al., [9]	To describe individual sleeping patterns and nocturnal cardiac autonomic activity of National team female soccer players during an international tournament.	Individually, players slept less than recommended amount (<7 h) on several days of the tournament, especially after 1 evening time match (n = 8; ranging between 6:00–6:54 h). Total sleep time CV ranged between 3.1 and 18.7%. However, all players presented good sleep quality (i.e., sleep efficiency ≥ 75%; individual range between 75–98%) on each day of the tournament.	The study highlights the substantial individual variability in sleep, suggesting the adoption of an individual approach to monitor sleep, training, and match loads and recovery to understand how players cope with highly demanding competitions better.
Costa et al., [10]	To highlight the substantial individual variability in sleep and HRV measures, suggesting the adoption of an individual approach to monitor sleep, training, and match loads and recovery to understand how players cope with highly demanding competitions better.	After 6 evening time training sessions, a higher number of players (17–22) slept less than 7 h/night, in contrast to the remaining days (i.e., match-days and rest days), but only 1–6 players had a sleep efficiency <75%. The CV for sleep duration and sleep efficiency ranged between 9–22% and 2–11%, respectively. A small negative within-subject correlation was found between TRIMP and sleep duration and sleep efficiency. A moderate and small negative within-subject correlation was found between s-RPE and sleep duration and sleep efficiency.	The study highlights the individual variability of sleep, indicating that sleep duration may be affected by training and match schedules and workloads.

s-RPE: session rated perceived exertion; CV: coefficient of variation.

**Table 7:** Results concerning sleep and injuries.

Study	Aim	Results	Findings
Chamari et al., [48]	To examine the effects over two consecutive years of the holy month of Ramadan on injury rates of a Tunisian top-level professional soccer team.	No significant differences between the three periods were observed for weekly mean training load, training strain, training duration, and Hooper’s Index (quality of sleep, and quantities of stress, delayed-onset muscle soreness, and fatigue). No significant difference in injury rates was observed between fasting and non-fasting players. Nevertheless, the rates of non-contact (6.8 vs. 0.6 and 1.1) and training overuse (5.6 vs. 0.6 and 0.5) injuries were significantly higher in RF than before or after Ramadan month.	Ramadan along with the corresponding changes in nutritional habits, sleeping schedule, and socio-cultural and religious events, significantly increased overuse and non-contact injuries in fasting players despite the fact that the training load, strain, and duration were maintained.
Gouttebarge et al., [29]	To explore the associations of severe musculoskeletal injuries (joint and muscles) and surgeries with symptoms of common mental disorders (distress, anxiety/ depression, sleeping disturbance, adverse alcohol behavior, smoking, adverse nutrition behavior) among male European professional soccer players.	Professional soccer players who had sustained three or more severe musculoskeletal injuries during their career were more than two times more likely to report sleeping disturbance (OR 2.3 and 95% CI 1.2–4.4) than professional soccer players who had not suffered from severe musculoskeletal injuries during their career. Professional soccer players who had sustained one or more severe joint injuries during their career were three to nearly four times more likely to report sleeping disturbance (OR 3.4 and 95% CI 1.9–6.2) than professional soccer players who had not suffered from severe joint injuries during their career.	European professional soccer players were 10–25% more likely to report symptoms of sleeping disturbance by every additional severe (joint or muscle) injury.
Kiliç et al., [49]	To explore the interaction between severe musculoskeletal time-loss injuries and symptoms of common mental disorders in professional soccer players over a 12-month period.	Symptoms of common mental disorders at baseline were not associated with the risk of severe musculoskeletal time-loss injury during the 12-month follow-up period, with relative risk of 0.6 (0.3–1.0) for sleep disturbance.	No relationship was found between symptoms of common mental disorders and the onset of severe musculoskeletal time loss injuries.
Krutsch et al., [50]	To investigate the factors influencing injuries in amateur soccer.	A total of 21.1% injuries happened during small-field soccer tournaments. The injury incidence of male players during match exposure was 469 per 1000 h soccer and significantly higher than in female players 313 (*p* = 0.025). Male players reported less and inadequate sleep the night before the tournament (*p* < 0.001).	Small-field tournaments in soccer have a high injury incidence. Male players have a higher injury incidence than female players and show a lack of sleep in the night before the tournament and poor warm-up performance on match day.
Laux et al., [19]	To examine the contribution of stress and recovery variables as assessed with the Recovery-Stress Questionnaire for Athletes (RESTQ-Sport) to the risk of injury in professional soccer players.	Overall, 34 traumatic injuries and 10 overuse injuries occurred. The recovery-related scale Sleep Quality (OR 0.53, *p* = 0.010) significantly predicted injuries in the month after the assessment.	The recovery-related scale sleep quality was a significant predictor implying that a lack of sleep or non-refreshing sleep also increases injury risk. It is suggested that a lack of recovery and sleep and high stress as well as a feeling of muscle strain and impending injury precede the injury and predict its occurrence.
Levitch et al., [51]	To provide a novel examination of the modifying role of sleep on the relationship between repetitive head impacts exposure and neuropsychological function in collegiate soccer players.	This sample had a high level of exposure to RHI, with a median count of 469 headers/year and 39 headers/two weeks, and they reported high levels of sleep disturbances, with over 50% of participants meeting criteria for “poor quality” sleep. With reduced sleep duration, a high level of recent heading exposure was related to worse sustained attention. However, with greater hours of sleep duration, heading exposure was related to preserved neuropsychological outcome in sustained attention.	Sleep may serve as a risk or protective factor for soccer players following extensive exposure to head impacts.
Nédélec et al., [18]	To examine the link between sleep and injury occurrence in an elite male soccer player competing in French League 1 and Union of European soccer Associations matches.	Three injuries were reported over the study period. Sleep onset latency, both in the single night (117 ± 43 min) and in the week (78 ± 50 min) before injury occurrence, was longer than pre-season baseline values (18 ± 13 min; ES: 3.1 and 1.6, respectively). Similarly, sleep efficiency in the single night (73 ± 7%) and the week (75 ± 7%) before injury occurrence was lower than baseline (90 ± 3%; ES: 3.2 and 2.8, respectively).	Sleep onset latency and efficiency were altered on the night and in the week before injury occurrence.
Silva et al., [17]	To investigate the relationship between sleep quality and quantity and injuries in elite soccer players. A secondary aim was to compare sleep-wake variables and injury characteristics.	The results indicated a moderate negative correlation between sleep efficiency and particular injury characteristics, including absence time, injury severity, and number of injuries. The linear regression analysis indicated that 44% of the total variance in the number of injuries (number) that can be explained by sleep efficiency, 24% of the total variance in the absence time after injury (days) that can be explained by sleep efficiency, and 47% of the total variance in the injury severity that can be explained by sleep efficiency.	Soccer players who exhibit lower sleep quality or non-restorative sleep show associations with increased amount and severity of musculoskeletal injuries.

RPE: rated perceived exertion; s-RPE: session rated perceived exertion; HR: heart rate; TL: training load; RF: Ramadan Fasting; RHI: repetitive head impacts; CI: confidence interval; OR: odds ration; ES: effect size.

## 4. Discussion

The discussion section covers the need for sleep, including the main evidence found about relationships between sleep patterns and athletic and match performance, training load, and injury occurrence in soccer players. Our review showed that soccer players are no exception for sleep inadequacy. Sleep restriction in soccer negatively affects athletic and match performance (Ajjimaporn et al. [27], Baati et al. [40]) and increases the number and severity of musculoskeletal injuries suffered by soccer players (Laux et al. [19], Silva et al. [17]).

On the other hand, inconsistent results were found regarding sleep’s relationships with athletic and match performance, training load, and athletic injuries in soccer players. For instance, Meckel et al. [36] found that the decrease in performance did not relate to sleeping hours during a fast in adolescent soccer players. Haddad et al. [30] found no significant correlations between training load and subjective sleep in all athletes. Owing to such inconsistencies, more research is required to understand these complex relationships better. In addition, future research should include analyses of objective and/or subjective measures of sleep associated with workloads, injuries, and performance outcomes during the competitive calendar in soccer to provide more insights into these associations.

### 4.1. Sleep and Athletic and/or Match Performance

Discerning the effects of sleep disturbance on athletic performance across studies is difficult, given the wide variety of study designs, populations, conditions, measurement tools, and reported outcomes. The specific mechanisms responsible for the associations between sleep and performance are not well-defined, and the effects vary depending on the task involved. Nevertheless, previous research has highlighted how common poor sleep quality is among soccer players. Most research has demonstrated that sleep deprivation inhibits performance in soccer (Ajjimaporn et al. [27], Abedelmalek et al. [33], Baati et al. [35], Fowler et al. [41]). For example, Ajjimaporn et al. [27] found that getting 3 h of sleep per night had negative effects on anaerobic performance tests, muscle strength, and fatigue in male college soccer players Therefore, a lack of sleep can limit the ability to train effectively to enhance strength and power. Because skill training requires optimal cognitive functioning for learning and memory to consolidate a new skill, efficiency of skill training each day to enhance competition performance will be limited because of sleep deprivation. The effects of sleep reduction have also been considered for specific exercises that stress different energy systems. For instance, in one study conducted by Fowler et al., [41], the intermittent sprint efforts involving anaerobic performance, sleep deprivation during simulated air travel resulted in slower sprint times. Ultimately, this outlines the need for athletes, globally independent of the predominant energy system used within their sport, to incorporate a sound sleep strategy to avoid any form of sleep loss.

### 4.2. Sleep and Training Load

Natural variations in training load imposed between and within weeks may disturb the biological rhythm of athletes and generate additional stress [8,9,10]. Moreover, the increase in training load at the origin of overreaching/overtraining development is often accompanied by changes in training and competition scheduling, which may influence the amount of time an athlete can spend in bed. For instance, Figueiredo and Costa et al. [8,9,10] found that the lowest and the highest workloads resulted in alterations in sleep durations during an international training camp in youth male soccer players and during a competitive two-week period in high-level female soccer players, showing also later bedtimes when training and matches were performed in the evening, close to bedtime sleep. The authors of the previous findings suggested that the negative associations between high training loads (such as those imposed during a soccer match) and sleep indices could be a result of overreaching and/or pro-inflammatory responses. In fact, when the balance between stress and recovery is disrupted, an abnormal training response can occur, and functional overreaching and overtraining can develop [2]. In contrast to the previous mentioned studies [8,9,10], Clemente et al. [42,43] found in both studies that sleep was also negatively correlated with training load, suggesting that congested periods, which involve increased stress and muscle soreness, are more likely to affect sleep quality than normal periods.

The use of extended sleeping time has been explored in past research within athletes who habitually endure sleep deprivation or experience significant sleep debt. In soccer, Watson et al. [46] found that sleep extension was significantly associated with improved fatigue, mood, and stress. In fact, a significant portion of the effect of training load on subjective well-being was because of the training load on sleep. As such, when designing training programs, practitioners need to consider the upcoming competitive schedules and should also be aware of the implications of workloads on sleep duration and fatigue levels. Poorly designed training programs and workloads may restrict the opportunity athletes have for sleep, which may limit recovery between training sessions and increase the risk of overreaching/overtraining.

### 4.3. Sleep and Injuries

Impaired or decreased sleep may increase risk of injury, but suffering an injury may also impair sleep quality, as shown by Gouttebarge et al. [29]. Moreover, Nédélec et al. [18] reported that poor sleep quality was related to the occurrence of musculoskeletal injury in soccer players. More recently, Silva et al. [17] found that soccer players who exhibit lower sleep quality or non-restorative sleep show associations with the increased amount and severity of musculoskeletal injuries. Even though such case studies are speculative, the physiological rationale for poor sleep to interfere with physiological recovery is evident, but evidence for this association between sleep and injury is lacking in the literature, especially in soccer. Sports injury is an emerging complex phenomenon and the risk factors of injury include nonlinear relationships between various factors such as the biomechanics, training characteristics, as well as psychological and physiological aspects. For instance, according to Laux et al.’s [19] findings, the greatest risk for injury seems to emerge from a simultaneous increase in training load and decrease in sleep duration; nevertheless, prospective randomized trials establishing that poor sleep quality precedes an injury could provide a more definitive answer. Hypothetically, and according to Silva et al. [17], poor sleep would lead to impaired reaction time and increased fatigue, predisposing the players to injury. Therefore, it is important to emphasize the care in relation to sleep and injuries of athletes so that it does not influence training, the recovery process, the sports performance of the athlete, or their team in competitions.

### 4.4. Study Limitations, Future Research, and Practical Implications

Some limitations should be considered according to the articles that were included in the present review. First, psychological, hormonal, and biomechanical aspects were not analyzed in depth, and the training environment was not controlled in most of the studies. Furthermore, the athletes should be evaluated in an integral and complex way, analyzing the relationships between various predictors of performance, workloads, and injury, and also be evaluated in relation to the duration and quality of sleep. Finally, there are very few high-quality, randomized controlled trials on athlete sleep, especially in soccer. In fact, the evidence showing the association between sleep, performance, workloads, and injuries are mostly observational, and for this reason causal interpretations should be avoided for now.

Educating players on sleep hygiene may improve general health and potentially improve performance. Beyond individual factors (e.g., timing of sleep, pre-sleep relaxation techniques), organizational factors (e.g., traveling, training schedules) should be considered in future research. Nevertheless, and according to the existing literature about sleep in soccer, to prevent poor sleep quality from having an impact on musculoskeletal injuries and/or reduced performance in soccer athletes, it is necessary to conduct a multifactorial assessment of the risks for the incidence of musculoskeletal injuries and performance impairment through an evaluation of athletes’ sleep, and to implement strategies which improve the sleep quality of athletes for an effective recovery to provide good performance.

## 5. Conclusions

Sleep quantity and quality interact with performance and may expose players to increased risk of injury, although more research is required. Strategies for improving sleep quantity and quality should consider training and competition schedules, traveling means and schedules, and psychosocial responses associated with the matches. School schedules and tests must be considered when coaching younger players. Sleep hygiene must be framed within a comprehensive set of strategies instead of relying on isolated data.

## Figures and Tables

**Table 1:** Eligibility criteria.

	Inclusion Criteria	Exclusion Criteria
Population	Soccer players from any age group, competitive level, or sex.	Sports other than soccer (e.g., futsal, beach football, basketball, rugby, Australian football), physical education students, general population.
Exposure	Players monitored for sleep quality and/or quantity.	Not controlled for sleep quality and/or quantity.
Comparator	Not mandatory. If available: comparisons between players with regular sleep (>7 h) and players identified with sleep depreviation and/or sleep loss (insomnia).	Sleep disorders other than sleep deprivation and/or sleep loss (e.g., sleep apnea, restless legs syndrom, narcolepsy).
Outcome	Relationships between objective and/or subjective sleep measures (e.g., number of hours of sleep; quality of sleep) and one of the following measures: (i) athletic performance (e.g., cardiorrespiratory fitness, anaerobic power, strength/power, mobility, speed, agility); (ii) match performance (e.g., cognitive performance, tactical/technical performance; match-running performance); (iii) training load (measures related to psychophysiological or physical load); and/or (iv) injury occurrence.	Other measures not related to athletic and match performance, training load, and injury occurrence (e.g., wellness measures). No effect of the exercise in sleep quality or quantity. Studies that analyzed the effect of exercise in sleep quality or quantity.
Other	Only original studies (not restricted to any language).	Abstracts, proceedings, letters, and other non-original studies.

## References

[1] Belenky G., Wesensten N.J., Thorne D.R., Thomas M.L., Sing H.C., Redmond D.P., Russo M.B., Balkin T.J. (2003). Patterns of performance degradation and restoration during sleep restriction and subsequent recovery: A sleep dose-response study. J. Sleep Res..

[2] Walsh N.P., Halson S.L., Sargent C., Roach G.D., Nédélec M., Gupta L., Leeder J., Fullagar H.H., Coutts A.J., Edwards B.J. (2021). Sleep and the athlete: Narrative review and 2021 expert consensus recommendations. Br. J. Sports Med..

[3] Tuomilehto H., Vuorinen V.-P., Penttilä E., Kivimäki M., Vuorenmaa M., Venojärvi M., Airaksinen O., Pihlajamäki J. (2017). Sleep of professional athletes: Underexploited potential to improve health and performance. J. Sports Sci..

[4] Kalkhoven J.T., Watsford M.L., Coutts A.J., Edwards W.B., Impellizzeri F.M. (2021). Training Load and Injury: Causal Pathways and Future Directions. Sports Med..

[5] Peake J.M., Kerr G., Sullivan J.P. (2018). A Critical Review of Consumer Wearables, Mobile Applications, and Equipment for Providing Biofeedback, Monitoring Stress, and Sleep in Physically Active Populations. Front. Physiol..

[6] Fullagar H.H.K., Skorski S., Duffield R., Hammes D., Coutts A.J., Meyer T. (2015). Sleep and Athletic Performance: The Effects of Sleep Loss on Exercise Performance, and Physiological and Cognitive Responses to Exercise. Sports Med..

[7] Fox J.L., Scanlan A.T., Stanton R., Sargent C. (2020). Insufficient Sleep in Young Athletes? Causes, Consequences, and Potential Treatments. Sports Med..

[8] Figueiredo P., Costa J., Lastella M., Morais J., Brito J. (2021). Sleep Indices and Cardiac Autonomic Activity Responses during an International Tournament in a Youth National Soccer Team. Int. J. Environ. Res. Public Health.

[9] Costa J., Figueiredo P., Nakamura F., Rago V., Rebelo A., Brito J. (2019). Intra-individual variability of sleep and nocturnal cardiac autonomic activity in elite female soccer players during an international tournament. PLoS ONE.

[10] Costa J.A., Figueiredo P., Nakamura F.Y., Rebelo A., Brito J. (2021). Monitoring Individual Sleep and Nocturnal Heart Rate Variability Indices: The Impact of Training and Match Schedule and Load in High-Level Female Soccer Players. Front. Physiol..

[11] Costa J.A., Brito J., Nakamura F.Y., Figueiredo P., Oliveira E., Rebelo A. (2019). Sleep patterns and nocturnal cardiac autonomic activity in female athletes are affected by the timing of exercise and match location. Chronobiol. Int..

[12] Ohayon M., Wickwire E.M., Hirshkowitz M., Albert S.M., Avidan A., Daly F.J., Dauvilliers Y., Ferri R., Fung C., Gozal D. (2017). National Sleep Foundation’s sleep quality recommendations: First report. Sleep Heal..

[13] Hirshkowitz M., Whiton K., Albert S.M., Alessi C., Bruni O., DonCarlos L., Hazen N., Herman J., Katz E.S., Kheirandish-Gozal L. (2015). National Sleep Foundation’s sleep time duration recommendations: Methodology and results summary. Sleep Health.

[14] Fullagar H.H.K., Skorski S., Duffield R., Julian R., Bartlett J., Meyer T. (2016). Impaired sleep and recovery after night matches in elite football players. J. Sports Sci..

[15] Sargent C., Halson S., Roach G.D. (2014). Sleep or swim? Early-morning training severely restricts the amount of sleep obtained by elite swimmers. Eur. J. Sport Sci..

[16] Lastella M., Roach G.D., Halson S.L., Sargent C. (2015). Sleep/wake behaviours of elite athletes from individual and team sports. Eur. J. Sport Sci..

[17] Silva A., Narciso F.V., Soalheiro I., Viegas F., Freitas L.S.N., Lima A., Leite B.A., Aleixo H.C., Duffield R., de Mello M.T. (2020). Poor Sleep Quality’s Association with Soccer Injuries: Preliminary Data. Int. J. Sports Physiol. Perform..

[18] Nédélec M., Leduc C., Dawson B., Guilhem G., Dupont G. (2019). Case Study: Sleep and Injury in Elite Soccer—A Mixed Method Approach. J. Strength Cond. Res..

[19] Laux P., Krumm B., Diers M., Flor H. (2015). Recovery–stress balance and injury risk in professional football players: A prospective study. J. Sports Sci..

[20] Souissi M., Abedelmalek S., Bou Dhiba D., Theodoros Nikolaidis P., Ben Awicha H., Chtourou H., Sahnoun Z. (2015). Morning caffeine ingestion increases cognitive function and short-term maximal performance in footballer players after partial sleep deprivation. Biol. Rhythm Res..

[21] Higgins J.P., Thomas J., Chandler J., Cumpston M., Li T., Page M.J., Welch V. (2019). Cochrane Handbook for Systematic Reviews of Interventions.

[22] Moher D., Liberati A., Tetzlaff J., Altman D.G. (2009). Preferred Reporting Items for Systematic Reviews and Meta-Analyses: The PRISMA Statement. PLoS Med..

[23] Collaboration C. Data Extraction Template for Included Studies. https://cccrg.cochrane.org/sites/cccrg.cochrane.org/files/public/uploads/det_2015_revised_final_june_20_2016_nov_29_revised.doc.

[24] Downs S.H., Black N. (1998). The feasibility of creating a checklist for the assessment of the methodological quality both of randomised and non-randomised studies of health care interventions. J. Epidemiol. Community Health.

[25] Simic M., Hinman R.S., Wrigley T.V., Bennell K.L., Hunt M.A. (2010). Gait modification strategies for altering medial knee joint load: A systematic review. Arthritis Care Res..

[26] O’Connor S.R., Tully M.A., Ryan B., Bradley J.M., Baxter G.D., McDonough S.M. (2015). Failure of a numerical quality assessment scale to identify potential risk of bias in a systematic review: A comparison study. BMC Res. Notes.

[27] Ajjimaporn A., Ramyarangsi P., Siripornpanich V. (2020). Effects of a 20-min Nap after Sleep Deprivation on Brain Activity and Soccer Performance. Int. J. Sports Med..

[28] Pallesen S., Gundersen H.S., Kristoffersen M., Bjorvatn B., Thun E., Harris A. (2017). The Effects of Sleep Deprivation on Soccer Skills. Percept. Mot. Skills.

[29] Gouttebarge V., Aoki H., Ekstrand J., Verhagen E.A.L.M., Kerkhoffs G.M.M.J. (2016). Are severe musculoskeletal injuries associated with symptoms of common mental disorders among male European professional footballers?. Knee Surg. Sports Traumatol. Arthrosc..

[30] Haddad M., Chaouachi A., Wong D.P., Castagna C., Hambli M., Hue O., Chamari K. (2013). Influence of fatigue, stress, muscle soreness and sleep on perceived exertion during submaximal effort. Physiol. Behav..

[31] Abbott W., Brett A., Watson A.W., Brooker H., Clifford T. (2020). Sleep Restriction in Elite Soccer Players: Effects on Explosive Power, Wellbeing, and Cognitive Function. Res. Q. Exerc. Sport.

[32] Aziz A.R., Che Muhamed A.M., Ooi C.H., Singh R., Chia M.Y.H. (2018). Effects of Ramadan fasting on the physical activity profile of trained Muslim soccer players during a 90-minute match. Sci. Med. Footb..

[33] Fowler P.M., Knez W., Thornton H.R., Sargent C., Mendham A.E., Crowcroft S., Miller J., Halson S., Duffield R. (2021). Sleep Hygiene and Light Exposure Can Improve Performance Following Long-Haul Air Travel. Int. J. Sports Physiol. Perform..

[34] Güvenç A. (2011). Effects of Ramadan Fasting on Body Composition, Aerobic Performance and Lactate, Heart Rate and Perceptual Responses in Young Soccer Players. J. Hum. Kinet..

[35] Hill D.W., Hill C.M., Fields K.L., Smith J.C. (1993). Effects of Jet Lag on Factors Related to Sport Performance. Can. J. Appl. Physiol..

[36] Meckel Y., Ismaeel A., Eliakim A. (2008). The effect of the Ramadan fast on physical performance and dietary habits in adolescent soccer players. Eur. J. Appl. Physiol..

[37] Zerguini Y., Kirkendall D., Junge A., Dvorak J. (2007). Impact of Ramadan on physical performance in professional soccer players. Br. J. Sports Med..

[38] Abedelmalek S., Boussetta N., Chtourou H., Souissi N., Tabka Z. (2014). Effect of partial sleep deprivation and racial variation on short-term maximal performance. Biol. Rhythm Res..

[39] Abedelmalek S., Chtourou H., Aloui A., Aouichaoui C., Souissi N., Tabka Z. (2013). Effect of time of day and partial sleep deprivation on plasma concentrations of IL-6 during a short-term maximal performance. Eur. J. Appl. Physiol..

[40] Baati H., Chtourou H., Moalla W., Jarraya M., Nikolaidis P.T., Rosemann T., Knechtle B. (2020). Effect of Angle of View and Partial Sleep Deprivation on Distance Perception. Front. Psychol..

[41] Fowler P., Duffield R., Vaile J. (2015). Effects of simulated domestic and international air travel on sleep, performance, and recovery for team sports. Scand. J. Med. Sci. Sports.

[42] Clemente F.M. (2018). Associations between wellness and internal and external load variables in two intermittent small-sided soccer games. Physiol. Behav..

[43] Clemente F.M., Mendes B., Nikolaidis P.T., Calvete F., Carriço S., Owen A.L. (2017). Internal training load and its longitudinal relationship with seasonal player wellness in elite professional soccer. Physiol. Behav..

[44] Selmi O., Gonçalves B., Ouergui I., Levitt D.E., Sampaio J., Bouassida A. (2019). Influence of Well-Being Indices and Recovery State on the Technical and Physiological Aspects of Play during Small-Sided Games. J. Strength Cond. Res..

[45] Watson A., Brickson S. (2018). Impaired Sleep Mediates the Negative Effects of Training Load on Subjective Well-Being in Female Youth Athletes. Sports Health Multidiscip. Approach.

[46] Watson A., Brickson S., Brooks A., Dunn W. (2017). Subjective well-being and training load predict in-season injury and illness risk in female youth soccer players. Br. J. Sports Med..

[47] Wilson D., Drust B., Reilly T. (2009). Is diurnal lifestyle altered during Ramadan in professional Muslim athletes?. Biol. Rhythm Res..

[48] Chamari K., Haddad M., Wong D.P., Dellal A., Chaouachi A. (2012). Injury rates in professional soccer players during Ramadan. J. Sports Sci..

[49] Kiliç Ö., Aoki H., Goedhart E., Hägglund M., Kerkhoffs G.M.M.J., Kuijer P.P.F.M., Waldén M., Gouttebarge V. (2018). Severe musculoskeletal time-loss injuries and symptoms of common mental disorders in professional soccer: A longitudinal analysis of 12-month follow-up data. Knee Surg. Sports Traumatol. Arthrosc..

[50] Krutsch V., Clement A., Heising T., Achenbach L., Zellner J., Gesslein M., Weber-Spickschen S., Krutsch W. (2020). Influence of poor preparation and sleep deficit on injury incidence in amateur small field football of both gender. Arch. Orthop. Trauma Surg..

[51] Levitch C.F., McConathey E., Aghvinian M., Himmelstein M., Lipton M.L., Zimmerman M.E. (2020). The Impact of Sleep on the Relationship between Soccer Heading Exposure and Neuropsychological Function in College-Age Soccer Players. J. Int. Neuropsychol. Soc..

